# The genome sequence of the long-horned nomad bee, *Nomada hirtipes *Pérez, 1884

**DOI:** 10.12688/wellcomeopenres.23163.1

**Published:** 2024-11-08

**Authors:** Steven Falk, University of Oxford and Wytham Woods Genome Acquisition Lab, Darwin Tree of Life Barcoding collective, Wellcome Sanger Institute Tree of Life Management, Samples and Laboratory team, Wellcome Sanger Institute Scientific Operations: Sequencing Operations, Wellcome Sanger Institute Tree of Life Core Informatics team, Tree of Life Core Informatics collective, Darwin Tree of Life Consortium

**Affiliations:** 1 Independent researcher, Kenilworth, England, UK

**Keywords:** Nomada hirtipes, long-horned nomad bee, genome sequence, chromosomal, Hymenoptera

## Abstract

We present a genome assembly from an individual female *Nomada hirtipes* (the long-horned nomad bee; Arthropoda; Insecta; Hymenoptera; Apidae). The genome sequence has a total length of 316.5 megabases. Most of the assembly (90.79%) is scaffolded into 16 chromosomal pseudomolecules. The mitochondrial genome has also been assembled and is 29.88 kilobases in length. Gene annotation of this assembly on Ensembl identified 11,693 protein-coding genes.

## Species taxonomy

Eukaryota; Opisthokonta; Metazoa; Eumetazoa; Bilateria; Protostomia; Ecdysozoa; Panarthropoda; Arthropoda; Mandibulata; Pancrustacea; Hexapoda; Insecta; Dicondylia; Pterygota; Neoptera; Endopterygota; Hymenoptera; Apocrita; Aculeata; Apoidea; Anthophila; Apidae; Nomadinae; Nomadini; *Nomada*; *Nomada hirtipes* Pérez, 1884 (NCBI:txid601583).

## Background

*Nomada hirtipes* Pérez, 1884, commonly known as the Long-horned Nomad Bee, is primarily found across Europe, including the UK, France, and Germany ( GBIF Secretariat, 2023). In Britain, there are records in southern and central England, with a patchy distribution from Devon to Kent and as far north as Derbyshire. It is absent from East Anglia, Scotland, Ireland, and the Channel Islands, and rare in Wales ( Falk, 2024).

Like all nomad bees, *N. hirtipes* is a kleptoparasite, specifically of *Andrena bucephala*. Females lay eggs in the host’s nest cell, where the larvae destroy the host egg or larva and consume the foodstore ( Falk, 2024). The habitats of *N. hirtipes* include scrubby grassland, old quarries, and churchyards, often with south-facing slopes, similar to those of its host.

The bee is univoltine, flying from late April to mid-June. Adults visit flowers such as Bogbean ( *Menyanthes trifoliata*), cow parsley ( *Anthriscus sylvestris*), cuckooflower ( *Cardamine pratensis*), and dandelions ( *Taraxacum* spp.) for nectar ( Else, 2024).

Both sexes of *N. hirtipes* have a slim build and long legs. Females are characterised by red markings on the lower face and tergite 2, with unusually long antennae bearing sparse, long hairs on the dorsal and posterior surfaces. Males exhibit a dark, furry thorax, a yellow lower face, and long antennae with a yellow underside to the scape ( Falk, 2024).

DNA sequencing is revealing complex patterns in *Nomada* species’ host associations, with some displaying host specificity and others using multiple hosts. Sequencing has identified cryptic species and distinct host races, showing the evolutionary plasticity of host choice. Full genome sequencing, such as the genome presented here through the Darwin Tree of Life project ( Blaxter *et al.*, 2022), may uncover further cryptic diversity within *Nomada* species ( Falk, 2024). This chromosomally complete genome sequence is based on a female specimen from Wytham Woods, Oxfordshire, UK.

## Genome sequence report

**Figure 1.**
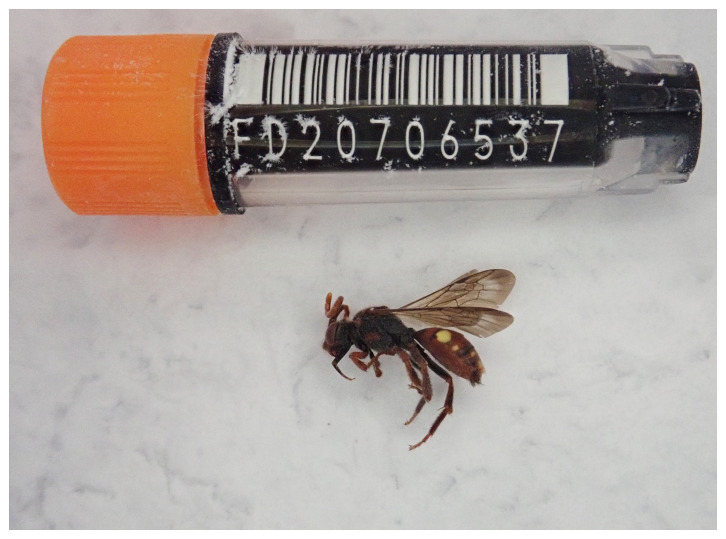
Photograph of the *Nomada hirtipes* (iyNomHirt1) specimen used for genome sequencing.

The genome was sequenced from an adult *Nomada hirtipes* ( Figure 1) collected from Wytham Woods, Oxfordshire, UK (51.76, –1.33). A total of 182-fold coverage in Pacific Biosciences single-molecule HiFi long reads was generated. Primary assembly contigs were scaffolded with chromosome conformation Hi-C data. Manual assembly curation corrected 64 missing joins or mis-joins, reducing the scaffold number by 37.20%, and increasing the scaffold N50 by 45.47%.

**Figure 2.**
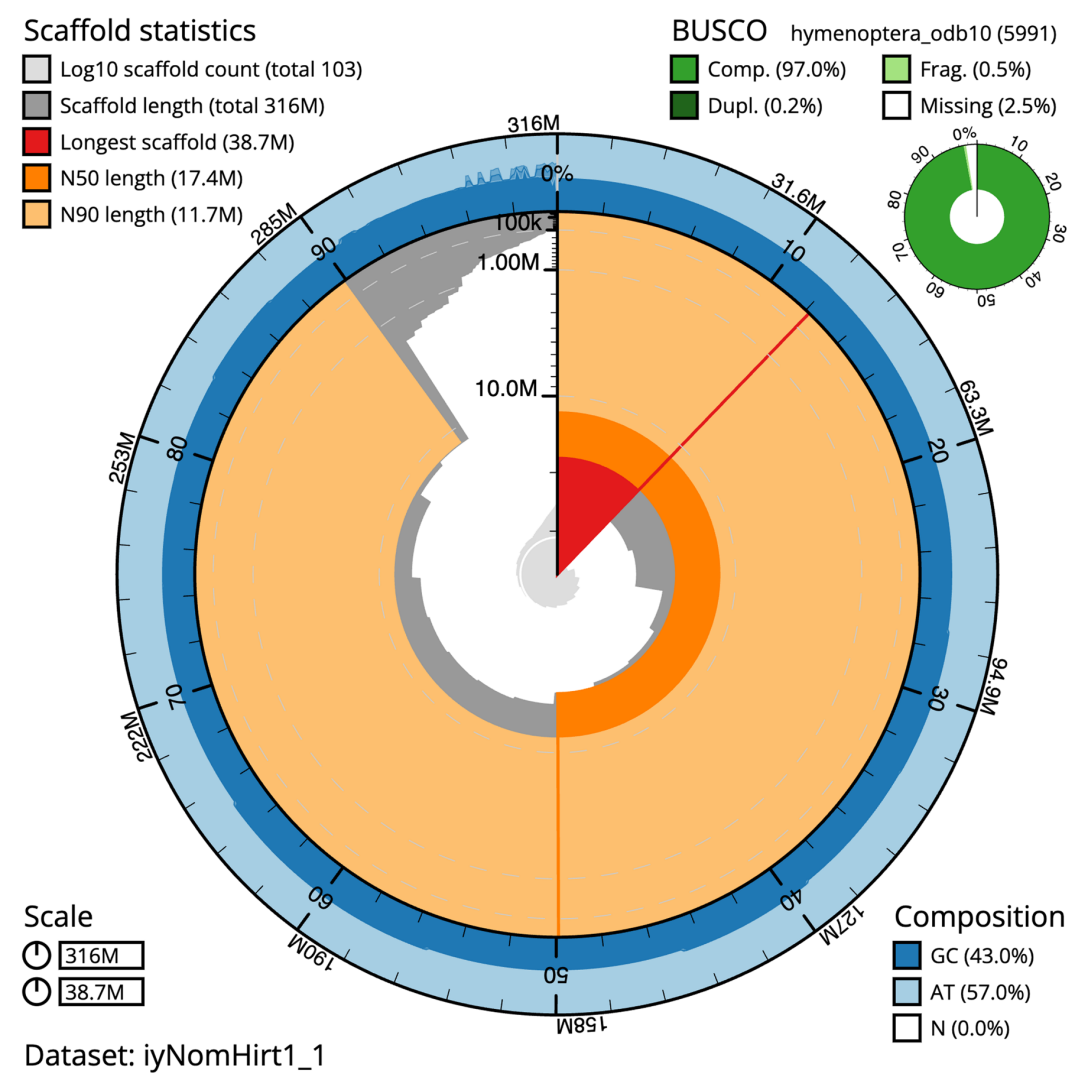
Genome assembly of *Nomada hirtipes*, iyNomHirt1.1: metrics. The BlobToolKit snail plot shows N50 metrics and BUSCO gene completeness. The main plot is divided into 1,000 size-ordered bins around the circumference with each bin representing 0.1% of the 316,499,700 bp assembly. The distribution of scaffold lengths is shown in dark grey with the plot radius scaled to the longest scaffold present in the assembly (38,659,845 bp, shown in red). Orange and pale-orange arcs show the N50 and N90 scaffold lengths (17,418,997 and 11,708,810 bp), respectively. The pale grey spiral shows the cumulative scaffold count on a log scale with white scale lines showing successive orders of magnitude. The blue and pale-blue area around the outside of the plot shows the distribution of GC, AT and N percentages in the same bins as the inner plot. A summary of complete, fragmented, duplicated and missing BUSCO genes in the hymenoptera_odb10 set is shown in the top right. An interactive version of this figure is available at https://blobtoolkit.genomehubs.org/view/Nomada%20hirtipes/dataset/iyNomHirt1_1/snail.

**Figure 3.**
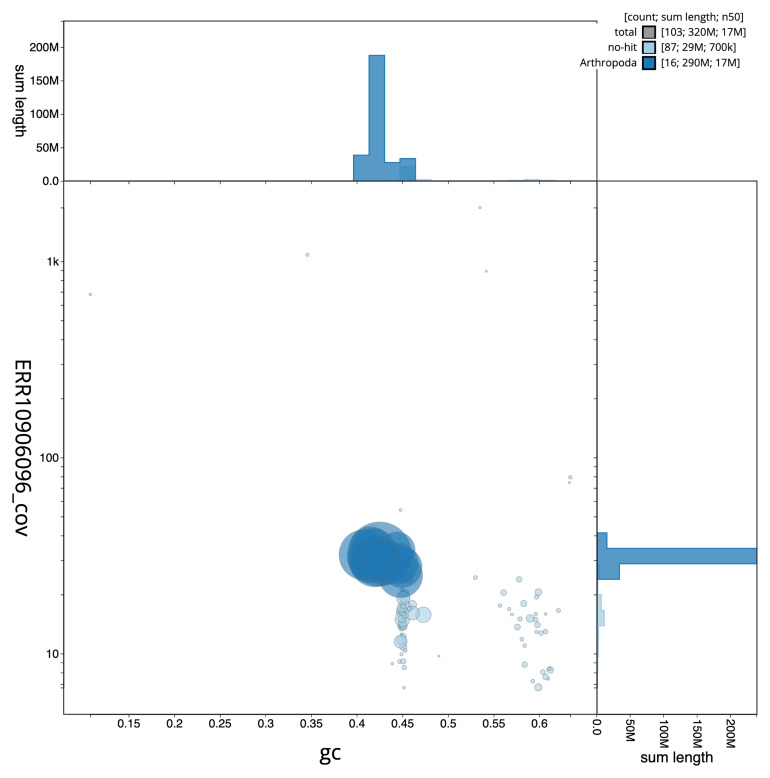
Genome assembly of *Nomada hirtipes*, iyNomHirt1.1: BlobToolKit GC-coverage plot. Sequences are coloured by phylum. Circles are sized in proportion to sequence length. Histograms show the distribution of sequence length sum along each axis. An interactive version of this figure is available at https://blobtoolkit.genomehubs.org/view/Nomada%20hirtipes/dataset/iyNomHirt1_1/blob.

**Figure 4.**
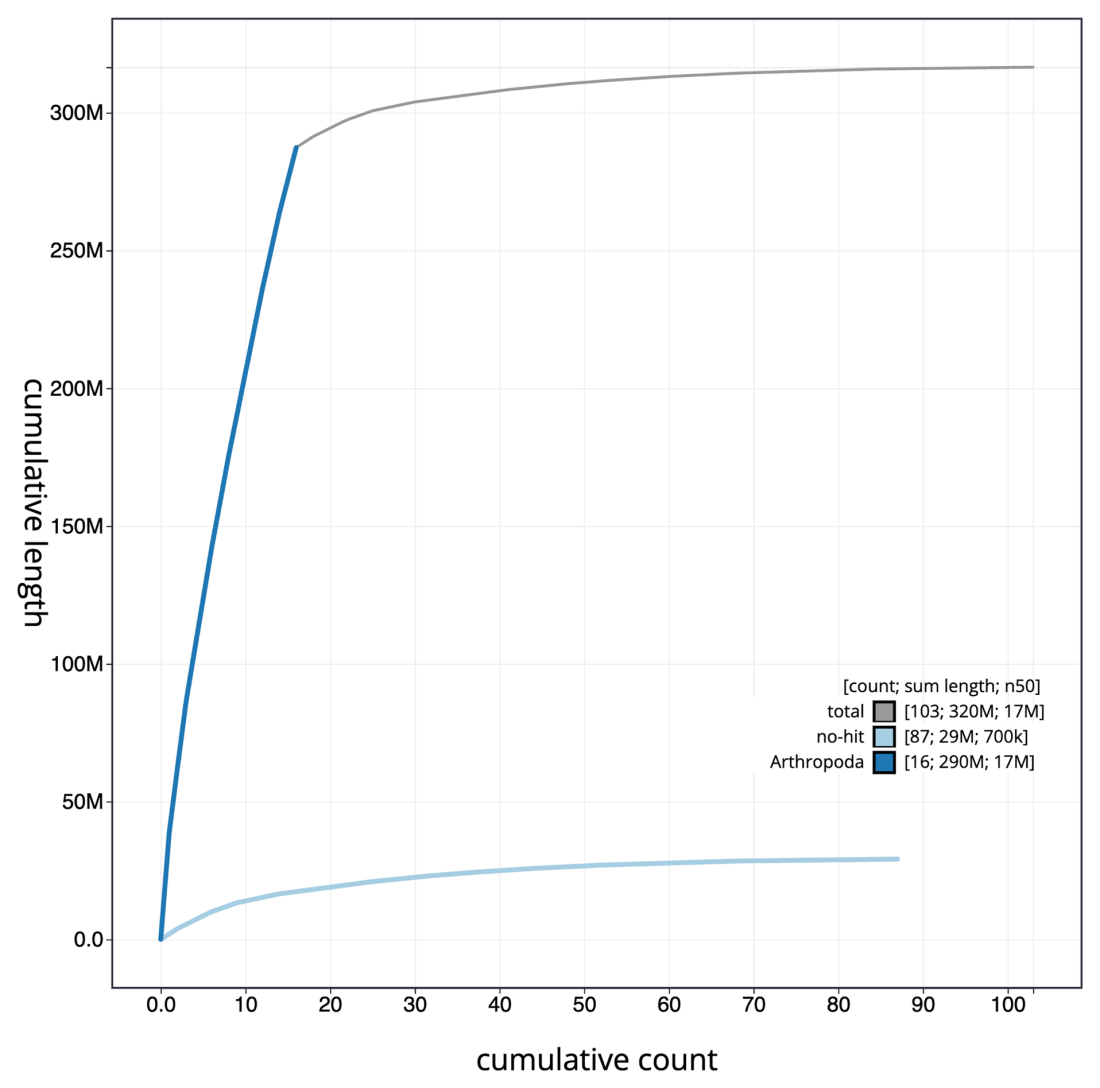
Genome assembly of *Nomada hirtipes* iyNomHirt1.1: BlobToolKit cumulative sequence plot. The grey line shows cumulative length for all sequences. Coloured lines show cumulative lengths of sequences assigned to each phylum using the buscogenes taxrule. An interactive version of this figure is available at https://blobtoolkit.genomehubs.org/view/Nomada%20hirtipes/dataset/iyNomHirt1_1/cumulative.

**Figure 5.**
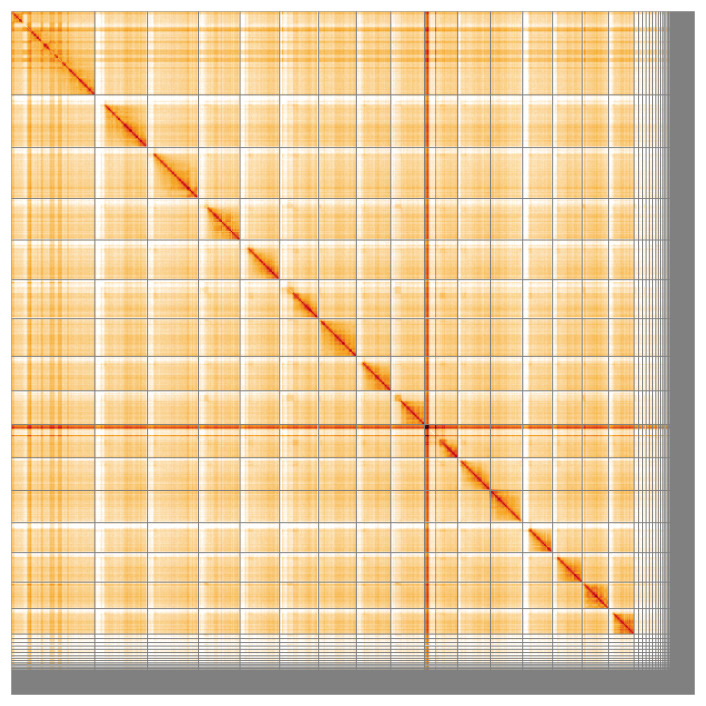
Genome assembly of *Nomada hirtipes* iyNomHirt1.1: Hi-C contact map of the iyNomHirt1.1 assembly, visualised using HiGlass. Chromosomes are shown in order of size from left to right and top to bottom. An interactive version of this figure may be viewed at https://genome-note-higlass.tol.sanger.ac.uk/l/?d=V9p7p8jQQjCsFu1kBqKx0A.

The final assembly has a total length of 316.5 Mb in 102 sequence scaffolds with a scaffold N50 of 17.4 Mb ( Table 1). The snail plot in Figure 2 provides a summary of the assembly statistics, while the distribution of assembly scaffolds on GC proportion and coverage is shown in Figure 3. The cumulative assembly plot in Figure 4 shows curves for subsets of scaffolds assigned to different phyla. Most (90.79%) of the assembly sequence was assigned to 16 chromosomal-level scaffolds. Chromosome-scale scaffolds confirmed by the Hi-C data are named in order of size ( Figure 5; Table 2). The order and orientation of contigs along Chromosome 10 is uncertain between 2.5 Mb and 7 Mb. While not fully phased, the assembly deposited is of one haplotype. Contigs corresponding to the second haplotype have also been deposited. The mitochondrial genome was also assembled and can be found as a contig within the multifasta file of the genome submission.

**Table 1.** Genome data for *Nomada hirtipes*, iyNomHirt1.1.

Project accession data		
Assembly identifier	iyNomHirt1.1	
Species	*Nomada hirtipes*	
Specimen	iyNomHirt1	
NCBI taxonomy ID	601583	
BioProject	PRJEB59954	
BioSample ID	Genome sequencing and Hi-C scaffolding: SAMEA10201329	
Isolate information	iyNomHirt1, female: whole organism (long read PacBio sequencing and Hi-C sequencing)	
Raw data accessions		
PacificBiosciences Sequel IIe	ERR10906096	
Hi-C Illumina	ERR10908633	
Genome assembly		
Assembly accession	GCA_951802735.1	
*Accession of alternate haplotype*	GCA_951802715.1	
Span (Mb)	316.5	
Number of contigs	216	
Number of scaffolds	102	
Longest scaffold (Mb)	38.66	
Assembly metrics *		*Benchmark*
Contig N50 length (Mb)	4.1	*≥ 1 Mb*
Scaffold N50 length (Mb)	17.4	*= chromosome N50*
Consensus quality (QV)	58.3	*≥ 40*
*k*-mer completeness	99.99%	*≥ 95%*
BUSCO **	C:97.1%[S:96.8%,D:0.3%], F:0.5%,M:2.4%,n:5,991	*S > 90%* *D < 5%*
Percentage of assembly mapped to chromosomes	90.79%	*≥ 90%*
Sex chromosomes	None	*localised homologous pairs*
Organelles	Mitochondrial genome: 29.88 kb	*complete single alleles*
Genome annotation of assembly GCA_951802735.1 at Ensembl		
Number of protein-coding genes	11,693	
Number of non-coding genes	3,857	
Number of gene transcripts	28,548	

* Assembly metric benchmarks are adapted from Rhie *et al.* (2021) and the Earth BioGenome Project Report on Assembly Standards September 2024. ** BUSCO scores based on the hymenoptera_odb10 BUSCO set using version 5.4.3. C = complete [S = single copy, D = duplicated], F = fragmented, M = missing, n = number of orthologues in comparison. A full set of BUSCO scores is available at https://blobtoolkit.genomehubs.org/view/Nomada%20hirtipes/dataset/iyNomHirt1_1/busco.

**Table 2.** Chromosomal pseudomolecules in the genome assembly of *Nomada hirtipes*, iyNomHirt1.

INSDC accession	Name	Length (Mb)	GC%
OX637916.1	1	38.66	42.5
OX637917.1	2	24.23	42.0
OX637918.1	3	23.53	41.0
OX637919.1	4	19.14	43.0
OX637920.1	5	18.3	42.0
OX637921.1	6	18.02	45.0
OX637922.1	7	17.42	42.0
OX637923.1	8	15.84	42.5
OX637924.1	9	15.54	45.0
OX637925.1	10	15.36	44.5
OX637926.1	11	15.13	41.0
OX637927.1	12	14.85	41.5
OX637928.1	13	13.74	41.5
OX637929.1	14	13.71	42.0
OX637930.1	15	12.17	44.5
OX637931.1	16	11.71	42.5
OX637932.1	MT	0.03	11.0

The estimated Quality Value (QV) of the final assembly is 58.3 with *k*-mer completeness of 99.99%, and the assembly has a BUSCO v completeness of 97.1% (single = 96.8%, duplicated = 0.3%), using the hymenoptera_odb10 reference set ( *n* = 5,991).

Metadata for specimens, barcode results, spectra estimates, sequencing runs, contaminants and pre-curation assembly statistics are given at https://links.tol.sanger.ac.uk/species/601583.

## Genome annotation report

The *Nomada hirtipes* genome assembly (GCA_951802735.1) was annotated at the European Bioinformatics Institute (EBI) on Ensembl Rapid Release. The resulting annotation includes 28,548 transcribed mRNAs from 11,693 protein-coding and 3,857 non-coding genes ( Table 1; https://rapid.ensembl.org/Nomada_hirtipes_GCA_951802735.1/Info/Index). The average transcript length is 10,424.32. There are 1.84 coding transcripts per gene and 6.26 exons per transcript.

## Methods

### Sample acquisition and DNA barcoding

An adult *Nomada hirtipes* (specimen ID Ox001529, ToLID iyNomHirt1) was collected from Wytham Woods, Oxfordshire (biological vice-county Berkshire), UK (latitude 51.76, longitude –1.33) on 2021-05-31 by netting. The specimen was collected and identified by Steven Falk (independent researcher) and preserved on dry ice.

The initial identification was verified by an additional DNA barcoding process according to the framework developed by Twyford *et al*. (2024). A small sample was dissected from the specimens and stored in ethanol, while the remaining parts were shipped on dry ice to the Wellcome Sanger Institute (WSI). The tissue was lysed, the COI marker region was amplified by PCR, and amplicons were sequenced and compared to the BOLD database, confirming the species identification ( Crowley *et al.*, 2023). Following whole genome sequence generation, the relevant DNA barcode region was also used alongside the initial barcoding data for sample tracking at the WSI ( Twyford *et al.*, 2024). The standard operating procedures for Darwin Tree of Life barcoding have been deposited on protocols.io ( Beasley *et al.*, 2023).

### Nucleic acid extraction

The workflow for high molecular weight (HMW) DNA extraction at the Wellcome Sanger Institute (WSI) Tree of Life Core Laboratory includes a sequence of core procedures: sample preparation and homogenisation, DNA extraction, fragmentation and purification. Detailed protocols are available on protocols.io ( Denton *et al.*, 2023b). The iyNomHirt1 sample was weighed and dissected on dry ice ( Jay *et al.*, 2023), and tissue from whole organism was homogenised using a PowerMasher II tissue disruptor ( Denton *et al.*, 2023a).

HMW DNA was extracted using the Automated MagAttract v2 protocol ( Oatley *et al.*, 2023a). DNA was sheared into an average fragment size of 12–20 kb in a Megaruptor 3 system ( Bates *et al.*, 2023). Sheared DNA was purified by solid-phase reversible immobilisation, using AMPure PB beads to eliminate shorter fragments and concentrate the DN ( Oatley *et al.*, 2023b). The concentration of the sheared and purified DNA was assessed using a Nanodrop spectrophotometer and Qubit Fluorometer using the Qubit dsDNA High Sensitivity Assay kit. Fragment size distribution was evaluated by running the sample on the FemtoPulse system.

### Hi-C preparation

Tissue from the iyNomHirt1 sample was processed at the WSI Scientific Operations core, using the Arima-HiC v2 kit. In brief, frozen tissue (stored at –80 °C) was fixed, and the DNA crosslinked using a TC buffer with 22% formaldehyde. After crosslinking, the tissue was homogenised using the Diagnocine Power Masher-II and BioMasher-II tubes and pestles. Following the kit manufacturer's instructions, crosslinked DNA was digested using a restriction enzyme master mix. The 5’-overhangs were then filled in and labelled with biotinylated nucleotides and proximally ligated. An overnight incubation was carried out for enzymes to digest remaining proteins and for crosslinks to reverse. A clean up was performed with SPRIselect beads prior to library preparation.

### Library preparation and sequencing

Library preparation and sequencing were performed at the WSI Scientific Operations core. Pacific Biosciences HiFi circular consensus DNA sequencing libraries were prepared using the PacBio Express Template Preparation Kit v2.0 (Pacific Biosciences, California, USA) as per the manufacturer's instructions. The kit includes the reagents required for removal of single-strand overhangs, DNA damage repair, end repair/A-tailing, adapter ligation, and nuclease treatment. Library preparation also included a library purification step using AMPure PB beads (Pacific Biosciences, California, USA) and size selection step to remove templates shorter than 3 kb using AMPure PB modified SPRI. DNA concentration was quantified using the Qubit Fluorometer v2.0 and Qubit HS Assay Kit and the final library fragment size analysis was carried out using the Agilent Femto Pulse Automated Pulsed Field CE Instrument and gDNA 165 kb gDNA and 55 kb BAC analysis kit. Samples were sequenced using the Sequel IIe system (Pacific Biosciences, California, USA). The concentration of the library loaded onto the Sequel IIe was in the range of 40–135 pM. The SMRT link software, a PacBio web-based end-to-end workflow manager, was used to set-up and monitor the run, as well as perform primary and secondary analysis of the data upon completion.

For Hi-C library preparation, DNA was fragmented to a size of 400 to 600 bp using a Covaris E220 sonicator. The DNA was then enriched, barcoded, and amplified using the NEBNext Ultra II DNA Library Prep Kit following manufacturers’ instructions. The Hi-C sequencing was performed using paired-end sequencing with a read length of 150 bp on an Illumina NovaSeq 6000 instrument.

### Genome assembly

The HiFi reads were first assembled using Hifiasm ( Cheng *et al.*, 2021) with the --primary option. Haplotypic duplications were identified and removed using purge_dups ( Guan *et al.*, 2020). The Hi-C reads were mapped to the primary contigs using bwa-mem2 ( Vasimuddin *et al.*, 2019). The contigs were further scaffolded using the provided Hi-C data ( Rao *et al.*, 2014) in YaHS ( Zhou *et al.*, 2023) using the --break option. The scaffolded assemblies were evaluated using Gfastats ( Formenti *et al.*, 2022), BUSCO ( Manni *et al.*, 2021) and MERQURY.FK ( Rhie *et al.*, 2020).

The mitochondrial genome was assembled using MitoHiFi ( Uliano-Silva *et al.*, 2023), which runs MitoFinder ( Allio *et al.*, 2020) and uses these annotations to select the final mitochondrial contig and to ensure the general quality of the sequence.

***Assembly curation***

The assembly was decontaminated using the Assembly Screen for Cobionts and Contaminants (ASCC) pipeline (article in preparation). Manual curation was primarily conducted using PretextView ( Harry, 2022), with additional insights provided by JBrowse2 ( Diesh *et al.*, 2023) and HiGlass ( Kerpedjiev *et al.*, 2018). Scaffolds were visually inspected and corrected as described by Howe *et al*. (2021). Any identified contamination, missed joins, and mis-joins were corrected, and duplicate sequences were tagged and removed. The process is documented at https://gitlab.com/wtsi-grit/rapid-curation (article in preparation).

***Evaluation of final assembly***

A Hi-C map for the final assembly was produced using bwa-mem2 ( Vasimuddin *et al.*, 2019) in the Cooler file format ( Abdennur & Mirny, 2020). To assess the assembly metrics, the *k*-mer completeness and QV consensus quality values were calculated in Merqury ( Rhie *et al.*, 2020). This work was done using the “sanger-tol/readmapping” ( Surana *et al.*, 2023a) and “sanger-tol/genomenote” ( Surana *et al.*, 2023b) pipelines. The genome evaluation pipelines were developed using nf-core tooling ( Ewels *et al.*, 2020) and MultiQC ( Ewels *et al.*, 2016), relying on the Conda package manager, the Bioconda initiative ( Grüning *et al.*, 2018), the Biocontainers infrastructure ( da Veiga Leprevost *et al.*, 2017), as well as the Docker ( Merkel, 2014) and Singularity ( Kurtzer *et al.*, 2017) containerisation solutions.

The genome was also analysed within the BlobToolKit environment ( Challis *et al.*, 2020) and BUSCO scores ( Manni *et al.*, 2021; Simão *et al.*, 2015) were calculated.

Table 3 contains a list of relevant software tool versions and sources.

**Table 3.** Software tools: versions and sources.

Software tool	Version	Source
BlobToolKit	4.2.1	https://github.com/blobtoolkit/blobtoolkit
BUSCO	5.3.2	https://gitlab.com/ezlab/busco
bwa-mem2	2.2.1	https://github.com/bwa-mem2/bwa-mem2
Cooler	0.8.11	https://github.com/open2c/cooler
Hifiasm	0.16.1-r375	https://github.com/chhylp123/hifiasm
HiGlass	44086069ee7d4d3f6f3f0012569789ec138f42b84aa44357826c0b6753eb28de	https://github.com/higlass/higlass
Merqury	MerquryFK	https://github.com/thegenemyers/MERQURY.FK
MitoHiFi	2	https://github.com/marcelauliano/MitoHiFi
Nextflow	23.04.0-5857	https://github.com/nextflow-io/nextflow
PretextView	0.2	https://github.com/wtsi-hpag/PretextView
purge_dups	1.2.3	https://github.com/dfguan/purge_dups
sanger-tol/genomenote	v1.0	https://github.com/sanger-tol/genomenote
sanger-tol/readmapping	1.1.0	https://github.com/sanger-tol/readmapping/tree/1.1.0
Singularity	3.9.0	https://github.com/sylabs/singularity
YaHS	yahs-1.1.91eebc2	https://github.com/c-zhou/yahs

### Genome annotation

The Ensembl Genebuild annotation system ( Aken *et al.*, 2016) was used to generate annotation for the *Nomada hirtipes* assembly (GCA_951802735.1) in Ensembl Rapid Release at the EBI. Annotation was created primarily through alignment of transcriptomic data to the genome, with gap filling via protein-to-genome alignments of a select set of proteins from UniProt ( UniProt Consortium, 2019).

### Wellcome Sanger Institute – Legal and Governance

The materials that have contributed to this genome note have been supplied by a Darwin Tree of Life Partner. The submission of materials by a Darwin Tree of Life Partner is subject to the **‘Darwin Tree of Life Project Sampling Code of Practice’**, which can be found in full on the Darwin Tree of Life website here. By agreeing with and signing up to the Sampling Code of Practice, the Darwin Tree of Life Partner agrees they will meet the legal and ethical requirements and standards set out within this document in respect of all samples acquired for, and supplied to, the Darwin Tree of Life Project.

Further, the Wellcome Sanger Institute employs a process whereby due diligence is carried out proportionate to the nature of the materials themselves, and the circumstances under which they have been/are to be collected and provided for use. The purpose of this is to address and mitigate any potential legal and/or ethical implications of receipt and use of the materials as part of the research project, and to ensure that in doing so we align with best practice wherever possible. The overarching areas of consideration are:

•   Ethical review of provenance and sourcing of the material

•   Legality of collection, transfer and use (national and international)

Each transfer of samples is further undertaken according to a Research Collaboration Agreement or Material Transfer Agreement entered into by the Darwin Tree of Life Partner, Genome Research Limited (operating as the Wellcome Sanger Institute), and in some circumstances other Darwin Tree of Life collaborators.

## Data Availability

European Nucleotide Archive: *Nomada hirtipes* (long-horned nomad bee). Accession number PRJEB59954; https://identifiers.org/ena.embl/PRJEB59954. The genome sequence is released openly for reuse. The *Nomada hirtipes* genome sequencing initiative is part of the Darwin Tree of Life (DToL) project. All raw sequence data and the assembly have been deposited in INSDC databases. The genome will be annotated using available RNA-Seq data and presented through the Ensembl pipeline at the European Bioinformatics Institute. Raw data and assembly accession identifiers are reported in Table 1.

## Author information

Members of the University of Oxford and Wytham Woods Genome Acquisition Lab are listed here: https://doi.org/10.5281/zenodo.7125292.

Members of the Darwin Tree of Life Barcoding collective are listed here: https://doi.org/10.5281/zenodo.4893703.

Members of the Wellcome Sanger Institute Tree of Life Management, Samples and Laboratory team are listed here: https://doi.org/10.5281/zenodo.10066175.

Members of Wellcome Sanger Institute Scientific Operations: Sequencing Operations are listed here: https://doi.org/10.5281/zenodo.10043364.

Members of the Wellcome Sanger Institute Tree of Life Core Informatics team are listed here: https://doi.org/10.5281/zenodo.10066637.

Members of the Tree of Life Core Informatics collective are listed here: https://doi.org/10.5281/zenodo.5013541.

Members of the Darwin Tree of Life Consortium are listed here: https://doi.org/10.5281/zenodo.4783558.
