# Supplementary material for: The genome sequence of the long-horned nomad bee, Nomada hirtipes Pérez, 1884
Source: Wellcome Open Res. 2024 Nov 8;9:665. [Version 1] doi: 10.12688/wellcomeopenres.23163.1 (PMC11809185; doi:10.12688/wellcomeopenres.23163.1)
Supplement: Peer review history

# Reviewer report: Reviewer response for version 1

**DOI:** 10.21956/wellcomeopenres.25507.r115747

**Published:** 2024-12-30

**Authors:** Manee M Manee

**Affiliations:** 1 King Abdulaziz City for Science and Technology, Riyadh, Saudi Arabia

The study presents the first genome assembly of the long-horned nomad bee, *Nomada hirtipes*, with a total length of 316.5 Mb and 90.79% of the sequence assigned to 16 chromosomal pseudomolecules. The mitochondrial genome was also assembled, and 11,693 protein-coding genes were annotated, showcasing a high-quality genome with 97.1% completeness as assessed by BUSCO. This assembly offers a foundation for further investigations into the bee's kleptoparasitic behavior and evolutionary diversity. I am pleased with the current form of the study.

Are sufficient details of methods and materials provided to allow replication by others?

Yes

Is the rationale for creating the dataset(s) clearly described?

Yes

Are the datasets clearly presented in a useable and accessible format?

Yes

Are the protocols appropriate and is the work technically sound?

Yes

Reviewer Expertise:

bioinformatics, genomics, evolutionary biology

I confirm that I have read this submission and believe that I have an appropriate level of expertise to confirm that it is of an acceptable scientific standard.

# Reviewer report: Reviewer response for version 1

**DOI:** 10.21956/wellcomeopenres.25507.r112186

**Published:** 2024-12-20

**Authors:** Ricardo Rodriguez-de-la-vega

**Affiliations:** 1 Écologie Systématique et Évolution, CNRS, Université Paris-Saclay, Paris, France

Comments on Falk and DToL consortium “The genome sequence of…”

 As of November 8th, this was the 14th true bee, the 111th Hymenoptera, the 776th Insecta, and approximately the 1000th overall entry in the Darwin Tree of Life's catalogue of chromosome-resolved genomes. Like most other entries in the catalogue, the assembly metrics are striking. The presentation follows the standard DToL genome report format, featuring informative and interactive snail, GC-coverage, and HiGlass plots, as well as assembly metrics, chromosome pseudomolecules, and software tables. I applaud the inclusion of the still unpublished 'Assembly Screen for Cobionts and Contaminants,' which is absent in most older DToL genome notes.

 As with all other DToL genome notes, readers may regret the lack of comparisons with other genomes, the almost complete absence of context regarding any potentially unique genomic features (although, in this case, the author does note that scaffold order in one chromosome region is uncertain), and the insufficient description of the 'second haplotype' contigs. As usual, this DToL genome note also includes the 'cumulative sequence plot,' which, in my opinion, is of limited use without any comparisons or attempts to identify the no-hit portion of the assembly (notably, all no-hits appear in unplaced scaffolds). Lastly, I still do not understand why the circles in the bottom-left of the snail plots are labeled 'Scale'.

Are sufficient details of methods and materials provided to allow replication by others?

Yes

Is the rationale for creating the dataset(s) clearly described?

Yes

Are the datasets clearly presented in a useable and accessible format?

Yes

Are the protocols appropriate and is the work technically sound?

Yes

Reviewer Expertise:

Comparative genomics, Evolutionary genomics, Bioinformatics

I confirm that I have read this submission and believe that I have an appropriate level of expertise to confirm that it is of an acceptable scientific standard.

# Reviewer report: Reviewer response for version 1

**DOI:** 10.21956/wellcomeopenres.25507.r115744

**Published:** 2024-12-19

**Authors:** Arun Arumugaperumal

**Affiliations:** 1 Rajalakshmi Engineering College, Chennai, Tamil Nadu, India

This is the first genome sequence report of the long-horned nomad bee, *Nomada hirtipes*. The genome assembly reported here is of length 316.5 Mb. They have assembled the mitochondrial genome and predicted 11,693 genes in the genome. The BUSCO genome completeness analysis shows 97% complete genome sequence. The data article can be indexed.

Are sufficient details of methods and materials provided to allow replication by others?

Yes

Is the rationale for creating the dataset(s) clearly described?

Yes

Are the datasets clearly presented in a useable and accessible format?

Yes

Are the protocols appropriate and is the work technically sound?

Yes

Reviewer Expertise:

Bioinformatics, Genomics

I confirm that I have read this submission and believe that I have an appropriate level of expertise to confirm that it is of an acceptable scientific standard.

# Reviewer report: Reviewer response for version 1

**DOI:** 10.21956/wellcomeopenres.25507.r115748

**Published:** 2024-12-17

**Authors:** Jason Charamis

**Affiliations:** 1 Foundation for Research and Technology - Hellas, Irákleion, Greece

The paper describes a reference genome assembly for the long-horned nomad bee *Nomada hirtipes*. The reported genome assembly is of good quality and its relevance is discussed well throughout the manuscript. I only have a few points that I think the authors should address before proceeding with the indexing of this report.  

 1. Rephrase here to improve readability and add a reference if possible:

 "DNA sequencing is revealing complex patterns in *Nomada* species’ host associations, with some displaying host specificity and others using multiple hosts. Sequencing has identified cryptic species and distinct host races, showing the evolutionary plasticity of host choice. Full genome sequencing, such as the genome presented here through the Darwin Tree of Life project ( Blaxter *et al.*, 2022), may uncover further cryptic diversity within *Nomada* species ( Falk, 2024). "

 Also explain better how a genome assembly will allow uncovering cryptic diversity with *Nomada* species.

 2. Here remove 'v' and replace 'single' with 'single-copy': 

 BUSCO v completeness of 97.1% (single = 96.8%, duplicated = 0.3%)

 3. Add 'base pairs' at the end of this sentence:

 The average transcript length is 10,424.32.

 4. Please include a BUSCO analysis of the predicted gene set (BUSCO on the protein mode) to have a quantitative assessment of how good the genome annotation is.

 5. Remove comma here 'joins, and' in this sentence:

 Any identified contamination, missed joins, and mis-joins were corrected, and duplicate sequences were tagged and removed.

Are sufficient details of methods and materials provided to allow replication by others?

Yes

Is the rationale for creating the dataset(s) clearly described?

Yes

Are the datasets clearly presented in a useable and accessible format?

Yes

Are the protocols appropriate and is the work technically sound?

Yes

Reviewer Expertise:

Computational and Comparative Genomics in arthropods

I confirm that I have read this submission and believe that I have an appropriate level of expertise to confirm that it is of an acceptable scientific standard, however I have significant reservations, as outlined above.

# Reviewer report: Reviewer response for version 1

**DOI:** 10.21956/wellcomeopenres.25507.r113183

**Published:** 2024-12-09

**Authors:** Alexis L Sperling

**Affiliations:** 1 University of Cambridge, Cambridge, England, UK

This report provides the genome sequence of the long-horned nomad bee. The introduction proves a nice background on this bee, a description of its unique features, and good rational for producing this assembly as part of the Darwin Tree of Life Project.

 Despite the uncertainty in chromosome 10, the mitochondria was sequences and the assembly is nearly phased. The snail plot and Table 1 give all of the importation information about the assembly quality and completeness. The assembly is of very high quality from the QV score of 58.3 and near 100% completeness. In figure 3/4 the BlobToolKit analysis indicates that there is little or no contamination. The Hi-C contact map in figure 5 shows that there is high confidence in the assemble scaffolds.

 Overall, it is a very good assembly that will be particularly interesting to those studying kleptoparasites.

Are sufficient details of methods and materials provided to allow replication by others?

Yes

Is the rationale for creating the dataset(s) clearly described?

Yes

Are the datasets clearly presented in a useable and accessible format?

Yes

Are the protocols appropriate and is the work technically sound?

Yes

Reviewer Expertise:

Parthenogenesis in Drosophila

I confirm that I have read this submission and believe that I have an appropriate level of expertise to confirm that it is of an acceptable scientific standard.
